# The neurochemical profile of the hippocampus in isoflurane-treated and unanesthetized rat pups

**DOI:** 10.1515/intox-2015-0017

**Published:** 2015-09

**Authors:** Petr N. Menshanov, Andrey E. Akulov

**Affiliations:** 1 Functional Neurogenomics Laboratory, Institute of Cytology and Genetics SBRAS, Russian Academy of Science, Novosibirsk State University MES RF, Novosibirsk, SFO, Russia; 2 Laboratory of Ecological Mammalian Genetics, Institute of Cytology and Genetics SBRAS, Russian Academy of Science, Novosibirsk, SFO, Russia

**Keywords:** neonatal rat, unanesthetized, isoflurane, hippocampus, magnetic resonance spectroscopy

## Abstract

*In vivo* study of cerebral metabolism in neonatal animals by high-resolution magnetic resonance spectroscopy (MRS) is an important tool for deciphering the developmental origins of adult diseases. Up to date, all in vivo spectrum acquisition procedures have been performed in neonatal rodents under anesthesia. However, it is still unknown if the inhaled anesthetic isoflurane, which is commonly used in magnetic resonance imaging studies, could affect metabolite levels in the brain of neonatal rats. Moreover, the unanesthetized MRS preparation that uses neonatal rodent pups is still lacking.

Here, a novel restraint protocol was developed for neonatal rats in accordance with the European Directive 2010/63/EU. This protocol shares the same gradation of severity as the protocol for non-invasive magnetic resonance imaging of animals with appropriate sedation or anesthesia. Such immobilization of neonatal rats without anesthesia can be implemented for MRS studies when an interaction between anesthetic and target drugs is expected. Short-term isoflurane treatment did not affect the levels of key metabolites in the hippocampi of anesthetized pups and, in contrast to juvenile and adult rodents, it is suitable for MRS studies in neonatal rats when the interaction between anesthetic and target drugs is not expected.

**LIST OF ABBREVIATIONS**
Ala: alanine
Asp: aspartate
Cho: choline compounds
Cr: creatine
GABA: gamma-aminobuytric acid
Glu: glutamine
Gln: glutamate
Ins: myoinositol
Lac: lactate
MRS: magnetic resonance spectroscopy
NAA: N-acetylaspartate
PCr: phosphocreatine
PD: ostnatal day
Tau: taurine

## Introduction

*In vivo* study of cerebral metabolism in neonatal animals by high-resolution magnetic resonance spectroscopy (MRS) is an important tool for deciphering the developmental origins of adult diseases that involve cognition and emotion, such as pain, addiction, depression, and psychiatric disorders (Xu *et al.*, 2013). For MRS study, reliable spectrum acquisition and reproducible measurements of brain metabolite levels are only possible if the subject cannot move. Unlike in humans, the immobilization of the animal for magnetic resonance study can be achieved only by anesthesia, by restraint or by training of the subject prior to spectrum detection (Xu *et al.*, 2013), and only the first two options are applicable for neonates (Tkác *et al.*, 2003; Tomiyasu *et al.*, 2013).

However, up to date, the immobilization system for magnetic resonance imaging studies exists for human neonates only (Tomiyasu *et al.*, 2013). Due to this fact, all *in vivo* spectrum acquisition procedures in neonatal rodents were performed under anesthesia, with the widespread adoption of the inhaled anesthetic isoflurane (Tkác *et al.*, 2003; Traudt *et al.*, 2012). Most drugs that are commonly used to anesthetize newborn rodents provide an inadequate level of immobilization or are associated with problems, such as excessively high mortality (Danneman & Mandrell, 1997). Only few anesthetics are allowed for use in neonates and these drugs are known to affect cerebral metabolic profiles in juvenile and adult rodents (Boretius *et al.*, 2013; Xu *et al.*, 2013). For example, a recent report by Boretius *et al.* (2013) demonstrated that inhaled anesthetics such as isoflurane dramatically raised the level of lactate in the brain of juvenile and adult mice. All pathological conditions investigated in rodents cannot be studied reliably in the presence of agents that may alter the levels of brain metabolites, and it is critical to know if commonly used and accepted inhaled anesthetics could induce such alterations in the neonatal brain (Xu *et al.*, 2013). However, the nonanesthetized MRS preparation that could be used in neonatal rodent pups is still lacking.

The goals of this study were to develop a restraint protocol to perform MRS experiments in immobilized unanesthetized rat pups and to investigate if the hippocampal metabolic profiles in neonatal pups determined by ^1^H MRS are affected by the commonly used inhaled anesthetic isoflurane.

## Materials and methods

### Animals

Wistar rats bred in the animal facilities at the Institute of Cytology and Genetics, Novosibirsk, were used. The colony was maintained under natural illumination at 22–24 °C with food and water available *ad libitum*. All procedures used on animals were in compliance with international European ethical standards (European Directive 2010/63/EU) and the Russian national instructions for the care and use of laboratory animals. The study was approved by the Bioethical Committee of the Institute of Cytology and Genetics SBRAS, Novosibirsk. All efforts were made to minimize animal suffering and to use only the number of animals necessary to produce reliable data. Adult female rats weighing 200–250 g were mated with males in the late afternoon. Vaginal smears were taken in the morning. The day of birth was defined as postnatal day 1 (PD1). On PD2 litters were culled to 8 pups. All experimental procedures were performed on PD3 male pups.

### Immobilization procedure

**Figure 1:**
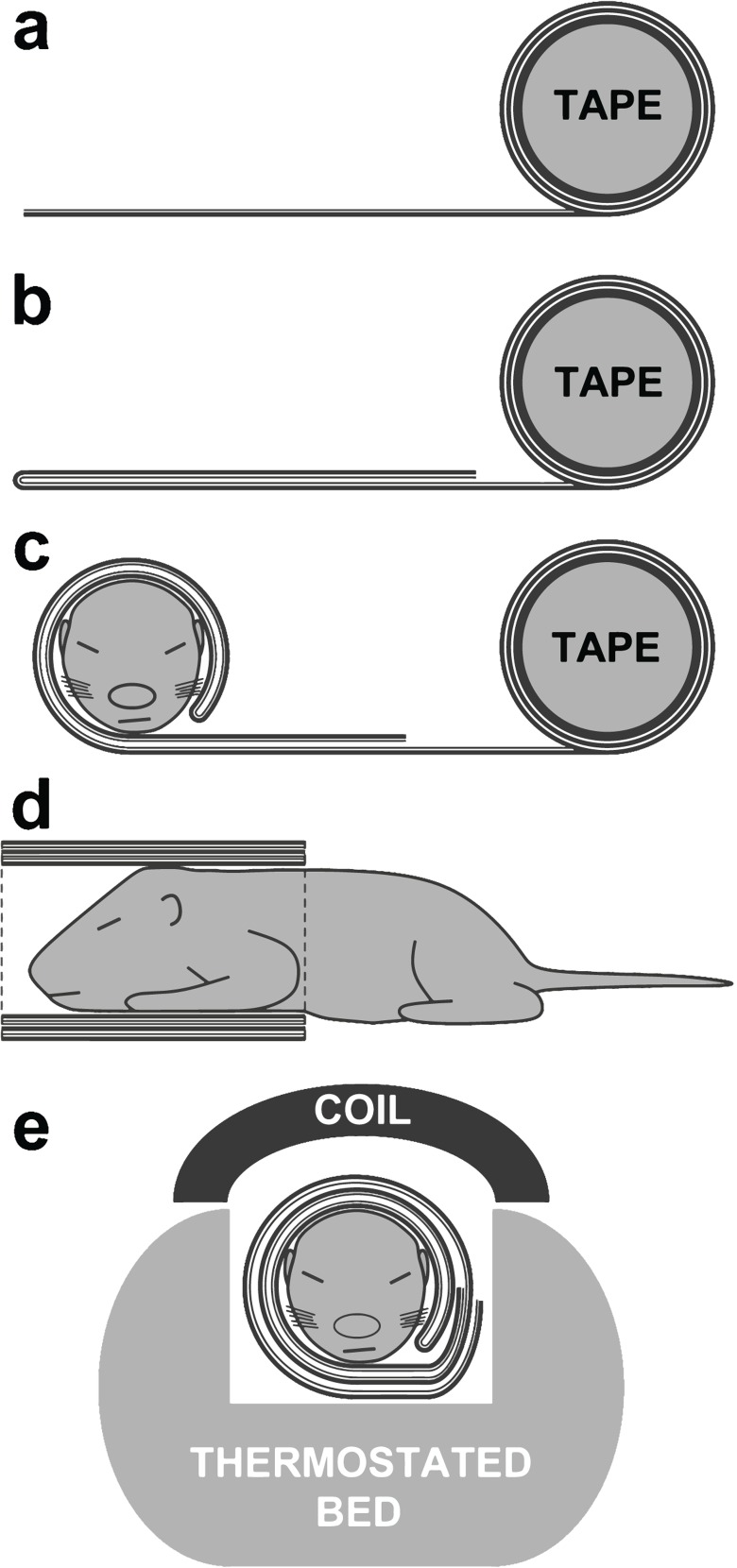
The immobilizing procedure for the neonatal rat pup. (see Materials and methods section for details).

The immobilization procedure required for spectrum acquisition in unanesthetized pups was developed in accordance with the European Directive 2010/63/EU. The standard single-sided adhesive tape (Figure 1a) was found to provide an excellent seal around the pups’ head and chest by restraining the movements of these body parts without any ventilatory impairment and with a minimal apparent discomfort for the animal. Before applying, the end of the tape was self-sticked to avoid the contact between the tape´s adhesive layer and the pup's skin (Figure 1b). The head and the chest of the experimental animal were placed on the self-sticked tape in a stretched position and wrapped by two consecutive rounds (Figure 1c,d). The end of the final round was self-sticked to fix the tape “tube” (Figure 1e). A respiratory pillow placed under the lower torso was used to monitor breathing frequency noninvasively (SA Instruments, Stony Brook, NY, USA). In preliminary studies, it was found that the sealed animal rested quietly in prone position after the first few minutes of restraining and eventually fell asleep, similarly to other immobilizing procedures used in neonatal rodents (Mortola, 1984; Tattersall & Milsom, 2003). The duration of the restraint was no more than 40 min. After all experimental procedures, the tape was removed without damaging the animal´s skin and vibrissae.

### ^1^H MR spectroscopy

All MRS experiments were performed on a horizontal 11.7T magnet (Bruker, BioSpec 117/16 USR, Germany) interfaced with a digital spectrometer operating at a resonant frequency of 500 MHz. The system is equipped with a 90-mm actively-shielded gradient set, with a maximum gradient strength of 740 mT/m. Radiofrequency excitation was accomplished with a 72-mm inner diameter linear birdcage coil and signal reception was achieved using a 25-mm inner diameter surface coil.

Isoflurane-treated pups (n=6) were anaesthetized through a mask by spontaneous inhalation of 2% isoflurane (300 mL/min) using Univentor 400 Anaesthesia Unit (Univentor Limited, Malta). Isoflurane was purchased from Sigma Chemical Company (St. Louis, USA). Untreated unanesthetized pups (*n*=7) were immobilized by tape as described earlier. The animals were placed in prone position and then slid into the magnet bore with a thermostated animal bed (Fig 1e). The body temperature was maintained at 36±1 °C by circulating heated water in the bed and was verified by a rectal thermosensor. Breath rate was monitored during the whole experiment. During MRS acquisition, the animals breathed freely. Recovery of isoflurane-treated pups after the interruption of anesthesia at the end of the procedure was rapid (<10 min).

Preformed high-resolution images were used to properly position the spectroscopic voxels for ^1^H MRS experiments. The voxel size (1.2×2.5×2.5 mm^3^) was the same in all MRS experiments. This voxel size allowed us to acquire MRS with a relatively high resolution in a reasonably short time of 15.5 min. All ^1^H MR spectra were acquired using a single voxel, stimulated echo acquisition mode spectroscopy sequence, with TE=3 msec, TM=20 msec, and TR=4000 msec, spectral width (4000 Hz), number of points=2048, and 230 signal averages. Prior to each spectral acquisition, localized voxel shimming was performed using the fast automatic shimming technique by mapping along projections technique. Shimming quality was estimated from the linewidth of the water peak in the spectra with unsuppressed water signal. For the water-suppressed spectra, water suppression was achieved by using variable pulse power and optimized relaxation delays sequence, and with proper adjustment of suppression pulse flip angles. For quantification of metabolites, a specialized computer program was designed. The basic concept used is that an *in vivo* ^1^H MR spectrum of a mixture is a linear combination of the *in vitro* spectra of the components, which is similar to that of the widely used LCModel. This method was described in detail by Moshkin *et al*. (2014).

**Figure 2:**
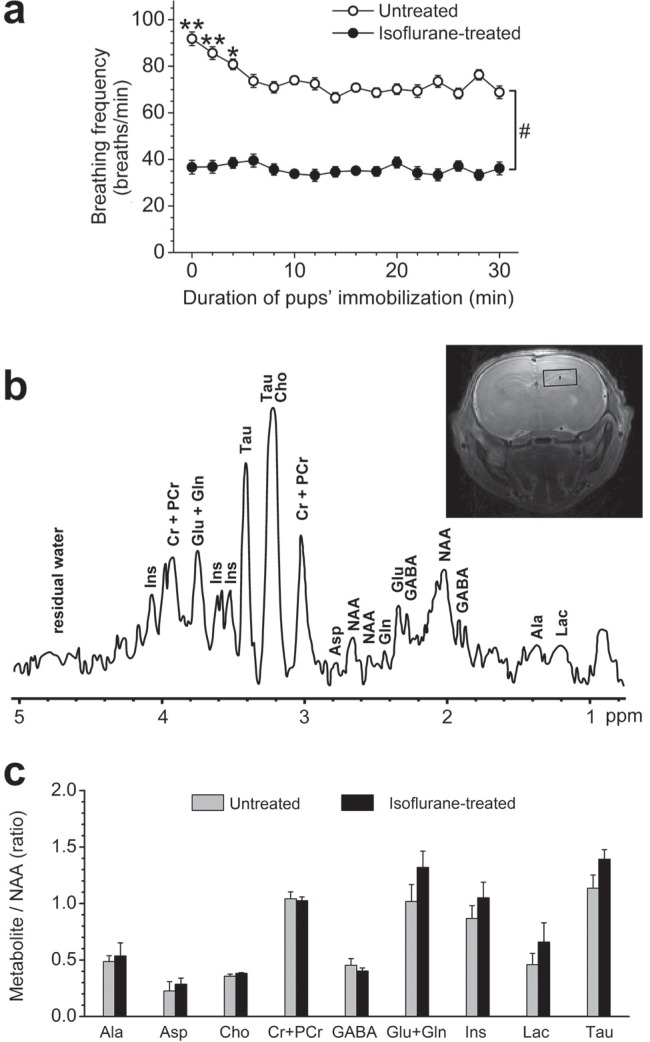
Breathing frequency of pups and neurochemical profile of the hippocampi of untreated and isoflurane-treated neonatal rats on PD3. **(a)** Breathing frequency of 3-day-old rat pups during restraint procedure and under isoflurane anesthesia. *p<0.05 vs breathing frequency of untreated rats in min 6–30. **p<0.001 vs breathing frequency of untreated rats in min 6–30. #*p*<0.001 vs breathing frequency of isoflurane-treated pups at the same time point. Data are presented as the means±SEM. **(b)** Representative *in vivo* short-echo-time 1H NMR spectrum at 11.7T in the hippocampus of untreated neonatal rat (TE=3 ms, TM=20 ms, TR=4 s, 230 scans, 7.5-μL volume) and RARE image of the PD3 rat brain with the volume of interest centered in the hippocampus and selected for 1H NMR spectroscopy studies. **(c)** Measured ratios of compounds (metabolite/NAA) in the hippocampi of untreated and isoflurane-treated neonatal rats. Ala – alanine; Asp – aspartate; Cho – choline compounds; Cr+PCr – creatine + phosphocreatine; GABA – gamma-aminobuytric acid; Glu+Gln – glutamine + glutamate; Ins – myo-inositol; Lac – lactate; Tau – taurine. Data are presented as the means±SEM. None of the ratios differed significantly between experimental groups.

### Data analysis

All data are represented as means±SEM. Data were analyzed by one way simple ANOVA (Treatment – simple factor), one-way repeated measures ANOVA (Minute – repeated measures factor) or two-way mix-design ANOVA (Treatment – simple factor; Minute – repeated measures factor). Statistical comparison of between-group differences was performed using the Fisher LSD post-hoc test. The results were considered significant at probability level less than 0.05.

## Results

All unanesthetized, by restraint immobilized pups had an increased breath frequency at the beginning of the test (Figure 2a; Minute – F_(15,90)_=9.73, *p*<0.0001). The breath rate of restrained pups fell to the baseline by minute 5–6 of monitoring and stayed stable up to the end of the procedure (Figure 2a). Isoflurane-treated animals had a stable breath frequency during the whole experiment (Figure 2a; Minute – F_(15,75)_=0.75, p=0.727), which was significantly lower in comparison with immobilized unanesthetized animals (Figure 2; Treatment – F(_1,11_)=2584.5, *p*<0.0001; Treatment*Minute – F_(15,165)_=4.43, *p*<0.0001). The mean rate of baseline breath frequency was 71±5 breaths/min for unanesthetized and 36±3 breaths/min for isofluranetreated pups (Figure 2a).

The developed procedure of immobilization was used to acquire proton spectra by MRS in the hippocampi of unanesthetized rat pups without the need for any postprocessing scheme to correct for possible motion during data acquisition. In the neonatal MR spectra, the peaks for nine target metabolites – Cr, PCr, Cho, NAA, Ins, Tau, Glu, Gln and Lac – were clearly observed and the peaks of three other compounds – GABA, Ala and Asp – were also detected (Figure 2b). The peaks of Glu and Gln as well as Cr and PCr strongly overlapped (Figure 2b). For this reason, in reference to other authors (Traudt *et al*., 2012), the data on these pairs of compounds were summed up and presented as the Glu+Gln and Cr+PCr integral data.

The established absolute NAA levels were not affected by isoflurane anesthesia (Untreated pups – 1.14±0.06 μmol/g wet weight; Isoflurane treated pups – 1.08±0.06 μmol/g wet weight; F_(1,11)_=0.46, p=0.510). There were no significant differences in the relative levels of any metabolite between the untreated unanesthetized and isoflurane-treated anesthetized pups (Figure 2c; Alanine – F_(1,11)_=0.18, p=0.680; Aspartate – F_(1,11)_=0.35, p=0.568; Choline compounds – F_(1,11)_=1.44, p=0.256; Creatine + Phosphocreatine – F_(1,11)_=0.06, p=0.819; GABA – F_(1,11)_=0.52, p=0.484; Glutamine + Glutamate – F_(1,11)_=2.05, p=0.180; Myo-Inositole – F_(1,11)_=1.08, p=0.320; Taurine – F_(1,11)_=2.94, p=0.114; Lactate – F_(1,11)_=1.11, p=0.314).

## Discussion

In this work, a novel system for restraint of neonatal rats was developed and successfully implemented for the first time to investigate the levels of metabolites in the hippocampi of unanesthetized neonatal rats by ^1^H MRS. It is well known that the restraint procedure is stressful for animals (King *et al*., 2005; Xu *et al*., 2013). The degree of animal discomfort during restraint could be measured by a number of invasive or noninvasive techniques (King *et al*., 2005; Zehendner *et al*., 2013). The monitoring of breathing rate during MRS acquisition is an obvious method of choice, as it allows continuous detection of the animal´s condition during experiment and is completely noninvasive (King *et al*., 2005).

All pups immobilized in our experiment by restraint had an increased breath frequency rate at the beginning of the test, similar to adult rats (Harris *et al*., 2005). Then, the increased breath rate of unanesthetized pups fell to the baseline and stayed stable up to the end of the procedure. It should be noted that the duration of the initial period with increased breath frequency was shorter in our pups than in the restrained adult rats (Harris *et al*., 2005). The mean rate of baseline breath frequency found in our unanesthetized pups was comparable with the previous estimations of this physiological measure reported by Tattersall and Milsom (2003) and Mortola (1984). The duration of the period of initial distress monitored in our study was in agreement with data reported by Zehender *et al*. (2013). These results indicated that each animal tested in our experiment experienced only a short-term initial discomfort during immobilization without any subsequent significant impairment of their well-being or general condition till the end of the procedure. Thus, the restraint procedure developed in this work was mild in terms of the European Directive 2010/63/EU and shared the same gradation of severity as the procedure of non-invasive MR imaging of animals with appropriate sedation or anesthesia.

The development of the restraint procedure allowed to detect metabolites in the hippocampi of unanesthetized neonatal rats by ^1^H MRS. Concentrations of all compounds determined in this study were within ranges reported for the neonatal rat brain previously (Florian *et al*., 1996; Tkác *et al*., 2003) and did not differ significantly between the untreated unanesthetized and isoflurane-treated anesthetized pups (Figure 2c). It should be noted that previous reports indicated a rather broad range of published metabolite levels in the immature brain regions during the first week of development (Florian *et al*., 1996; Miller *et al*., 2000; Tkác *et al*., 2003). In our study, relatively low Tau/NAA ratios were found in the hippocampi of both untreated and isoflurane-treated animals in comparison with values reported in the previous non-MR studies (Figure 2c; Miller *et al*., 2000). These findings are not surprising as the taurine levels are usually underestimated in the developing brain by *in vivo* MRS studies (Miller *et al*., 2000; Tkác *et al*., 2003). The underestimation of the Tau/NAA ratio probably occurred due to the partial interference of the taurine peak at 3.25 ppm with other peaks related to choline compounds, phosphorylethanolamine and myo-inositol (Tkác *et al*., 2003).

It is well established that anesthesia lowers the breath frequency (Zehendner *et al*., 2013). Indeed, our isoflurane-treated animals had a stable, reduced breath rate in comparison with immobilized unanesthetized animals. The decreased ventilation is usually associated with impairment of oxygen delivery to brain tissue and alterations in the levels of metabolites related to energy metabolism, such as lactate (Zehendner *et al*., 2013). It cannot be excluded that such mechanism might be responsible for lactate upregulation found in the brain of isoflurane-treated juvenile and adult mice (Boretius *et al*., 2013). However, neonatal rats are known to be wellresistant to hypoxic conditions up to PD4–5 (Mach *et al*., 2009) and the observed reduction in the breath rate of our isoflurane-treated pups did not affect lactate levels in their hippocampus, as shown by MRS quantification (Figure 2c).

In conclusion, a novel restraint protocol was developed for neonatal rodents in this work. This protocol shares the same gradation of severity as the protocol for non-invasive MR imaging of animals with appropriate sedation or anesthesia. Due to this fact, the developed protocol can be implemented for MRS studies in neonatal rats when an interaction between the anesthetic and the target drug is expected. Immobilization of neonatal rodents without sedation could also be applied for functional magnetic resonance imaging studies. It was also found that shortterm isoflurane anesthesia did not affect the levels of key metabolites in the neonatal hippocampus. Our data provide evidence that, in contrast to juvenile and adult rodents, short-term isoflurane anesthesia can be used for MRS studies in neonates when interaction between the anesthetic and the target drug is not expected.
